# Abuses in psychiatric care: The shameful story of the Lake Alice Child and Adolescent unit in Aotearoa New Zealand

**DOI:** 10.1177/00048674231193381

**Published:** 2023-08-07

**Authors:** Susanna Every-Palmer, Oliver Sutherland

**Affiliations:** 1 Department of Psychological Medicine, University of Otago, Wellington, Wellington, New Zealand; 2 Auckland Committee on Racism and Discrimination, Member of the Royal Commission Forum, Nelson, New Zealand

‘The story of the Lake Alice Child and Adolescent unit is a shameful chapter in the history of Aotearoa New Zealand, which must be faced head-on without excuses or explanations, but with a determination to accept the injustice, make proper amends and ensure this tragedy never happens again’ (p. 27, Abuse in Care–Royal Commission of Inquiry, 2022).

In Aotearoa, New Zealand, a Royal Commission of Inquiry is underway, investigating abuses that occurred in State and faith-based care, including psychiatric care, between 1950 and 1999. The full report and recommendations are expected next year. However, in December 2022, the Commission published an early case study report, *Beautiful Children* (‘the report’), which specifically investigated the Lake Alice Child and Adolescent Psychiatric Unit, as had been recommended by the United Nations. More than 324 pages, the harrowing experiences of the young people admitted to Lake Alice during the unit’s short existence in the 1970s are meticulously catalogued (Abuse in Care–Royal Commission of Inquiry, 2022). The report details the myriad ways young people were hurt by psychiatric staff and how they were disbelieved and dismissed by State agencies when they reported their experiences.

The worst perpetrator of the abuse was the unit’s head psychiatrist, Selwyn Leeks, who was a Fellow of the Royal Australian and New Zealand College of Psychiatrists (RANZCP) until last year, and who practised and caused harm in both countries. But the culpability falls much more broadly than at the feet of a lone rogue psychiatrist; the Lake Alice story is also a story of a toxic culture, systems failures, staff complicity, institutional racism, and a litany of failings by State agencies.

The authors read the report from two different perspectives. Oliver Sutherland is a retired scientist and a lifelong campaigner of social justice. Now in his early 80s, Oliver was a founding member of the Auckland Committee on Racism and Discrimination (ACORD) in the 1970s. He has known about the horrors of Lake Alice for 46 years. In fact, he and his ACORD colleagues were the first whistle-blowers, and he is now a member of Royal Commission Forum, a group monitoring the work of the Abuse in Care Inquiry. Sadly, the Commission’s findings no longer shocked him – they gave voice to truths he had known for years.

Susanna Every-Palmer is a psychiatrist in her 40s and an academic. To her shame, prior to the Royal Commission Inquiry, she had not known about the atrocities at Lake Alice despite these occurring during her lifetime and being in the public domain for many years; an ignorance she shared with many colleagues. The Commission’s findings profoundly shocked her – they gave voice to truths she did not want to believe could be possible in New Zealand.

Wilful blindness cannot be the response. To say the report makes for difficult reading is an understatement, but everyone working in mental health in New Zealand and Australia should be aware of its contents. Here, we summarise what happened at Lake Alice and discuss the implications for modern psychiatry.

## What was the Lake Alice Child and Adolescent Unit?

The Lake Alice Child and Adolescent Unit was a psychiatric unit in rural Manawatū, adjoining other facilities, including an adult maximum secure unit. It was located almost 40 km from Whanganui and 50 km from Palmerston North, making it logistically difficult for many families to visit the children held there. The unit opened in 1972 and over the next 6 years admitted between 400 and 450 children and adolescents, with a median age at first admission of 13 years. Admissions were stopped in 1978 under the spectre of allegations of mistreatment and abuse, and the unit permanently closed in 1980.

Despite it being a psychiatric facility, most of the children admitted to Lake Alice did not in fact have a mental illness; Department of Social Welfare records show that 60% of admissions were for ‘behavioural’ problems. Many of these children came from disadvantaged or marginalised communities often with significant histories of childhood adversity and trauma. Māori children were over-represented. About 41% of admissions from social welfare residences and about 29% of admissions from homes with social welfare files involved Māori children (Abuse in Care–Royal Commission of Inquiry, 2022). For comparison, the relative percentage of the general population recorded as having Māori ethnicity in 1975 was 8% (NZ Department of Statistics, 1975).

Dr Selwyn Leeks was the head psychiatrist of the unit from when it opened until he left, precipitously and under a dark cloud, although not formally sanctioned, in 1977. For the next quarter of a century, Dr Leeks continued practicing as a child psychiatrist in Melbourne, but not without further controversy, as we later explain.

## Who did the Commission hear from and what did they find?

More than a hundred survivors of the Lake Alice unit and other witnesses gave evidence to the Commission during the public hearing in June 2021. The Commission noted they repeatedly heard accounts of children running way from Lake Alice, only to be apprehended and returned to the unit. At the time, no one believed them if they tried to explain what it was they were running from. The Commission found many reasons to desire escape, summarising

in the almost eight years the unit operated, Dr Leeks and the staff at Lake Alice inflicted, or oversaw, serious abuse – some amounting to torture – in what quickly became a culture of mistreatment, physical violence, sexual and emotional abuse, neglect, threats, degradation and other forms of humiliation. (p. 24, Abuse in Care–Royal Commission of Inquiry, 2022).

## The use of electric shocks at Lake Alice

Dr Leeks was a frequent user of an old electrotonus electroconvulsive therapy (ECT) machine at his unit. In response to concerns raised at the time regarding his practices, he stated he always gave treatment therapeutically and not as a punishment. This was a lie.

In terms of understanding how egregious Dr Leeks’ practices were according to any standards, even those of the day, it is worth considering a brief history of the use of ECT in New Zealand. In the first decade after its introduction here in the 1940s, ECT was sometimes administered in an ‘unmodified’ form, meaning a patient remained conscious and no muscle relaxant was used (Abuse in Care–Royal Commission of Inquiry, 2022). This could be dangerous and painful, with risks including fractures, severe pain, or choking. From the 1950s onward, unmodified ECT was phased out and treatment was instead administered with concomitant muscle relaxant and general anaesthetic (Abuse in Care–Royal Commission of Inquiry, 2022). In an investigation in the 1970s, journalist Peter Trickett examined ECT in five psychiatric hospitals in the Auckland/Waikato region. He found, unlike Lake Alice, none of these hospitals used ECT for children. For adults, the use of unmodified ECT without anaesthesia was only ever considered at Oakley Hospital, and only in extreme circumstances (Trickett, 1977 as cited by Sutherland, 2020).

While ECT appears to have sometimes been used at Lake Alice in a conventional sense, much of the time, the ECT machine was deployed for a purpose Dr Leeks termed ‘aversion therapy’ and the United Nations termed ‘torture’ (United Nations Committee Against Torture, 2020). This entailed giving electric shocks to children to ‘disincentivise’ unwanted behaviour. These shocks were administered without anaesthetic, without muscle relaxant, at high doses, at unconventional intervals, with aberrantly positioned electrodes, and for reasons that had nothing to do with mental illness.

In breach of the law, consent was seldom sought from patients or their legal guardians.

Let us first consider some of the indications for which shocks were administered. Lake Alice medical records state these included ‘passing wind’, being ‘anti-social’, being picky about food, ‘being in a world of his own’, ‘showing off in front of the girls in class’, ‘annoying others during work periods’, and ‘being argumentative’ (p. 168, Abuse in Care–Royal Commission of Inquiry, 2022).

Next was the modality of administration. Muscle relaxants and anaesthetic were often eschewed. As well as the temples, Dr Leeks frequently applied electrodes to a child’s torso or limbs and the Commission heard about at least 15 young people having shocks delivered directly to their breasts, groin, or genitals causing excruciating pain, ‘like hot needles going into your testicles’ (p. 88, Abuse in Care–Royal Commission of Inquiry, 2022). Sometimes young people were required to administer shocks to their peers or were forced to watch while others were shocked. Survivors repeatedly told the Commission of urinating or defecating in anticipatory terror, of screaming in pain, and of hearing the screams of other children reverberate throughout the unit.

**Table:** 

My arms and legs flailed about in agony. You just wouldn’t believe how bad the pain was. They would call your name while you were having lunch, and that meant you had to stay. We were taken to a day room and locked in, so we couldn’t escape. I was often so scared that I soiled myself . . . I tried to run away once, so Dr Leeks gave me electric shocks on my feet to teach me a lesson. The pain was worse because it ran up into the rest of my body . . . I was also raped by another patient repeatedly and when I told the staff, they didn’t believe me – and punished me for “lying” by giving me more electric shocks. My records show I was even given shocks for “showing off in front of the girls”. You just got what was given to you, you had no control over anything. Account of survivor BN (p. 108, Abuse in Care–Royal Commission of Inquiry, 2022)

Dr Leeks’ misuse of the ECT machine was never legitimate psychiatric treatment, it was serious assault. It is hard to understand why this was not reported by other staff or colleagues, but the institutional culture at the unit appears to have inured staff to abusive practices. Most staff seemed to have accepted that this was just the way things were. Some participated willingly, some passively, some spoke up, but tentatively. A nurse recalled voicing concerns and having the precarity of their residence in hospital accommodation pointed out by Dr Leeks and being instructed not to question his clinical judgement again. This nurse explained to the Commission, ‘I think that Dr Leeks put himself above being personally affected by administering [electric shocks], and in so doing, failed to recognise the development of his own sadism and that of the staff that worked for him’ (p. 84, Abuse in Care–Royal Commission of Inquiry, 2022).

## Other forms of abuse

A punitive culture permeated the unit. Staff training and resourcing were inadequate and prejudiced attitudes abounded, including systemic racism, ableism, and homophobia. The care provided was insensitive to the trauma many children had experienced and instead they were seen as merely naughty and in need of firm discipline. As well as the so-called electric shock ‘aversion therapy’, paraldehyde was injected intramuscularly by nursing staff in deliberate attempts to cause pain to deter bad behaviour. Survivors told the inquiry paraldehyde injections were given liberally in response to things such as kicking a ball at a window, theft, fighting, smoking, and throwing apples. Other punitive measures included children being secluded for long periods, with survivor Leoni McInroe giving evidence on one occasion she was locked in solitary confinement for 21 continuous days.

The Commission also found sexual abuse was common, with perpetrators including staff members, adult patients, and other patients in the unit. One nurse was convicted and imprisoned in 1972 for sexually assaulting a child but other staff implicated in multiple allegations of serious sexual abuse were never charged. Survivors were often not believed, and when it was acknowledged that abuse had occurred, allegations were not always referred to the Police. Dr Pugmire, the Lake Alice medical superintendent later explained contraceptives were routinely administered to teenage girls on the unit to ‘avoid the embarrassment of having to explain to the respective mothers that they have been fertilised by a passing criminal psychopath’ (p. 128, Abuse in Care–Royal Commission of Inquiry, 2022).

Overall, the impact of the abuse of children and young people at Lake Alice, whether directly experienced or witnessed, has had severe lifelong consequences for many survivors’ mental and physical health. Some survivors still bear the physical stigmata and symptoms from this abuse, including scars from electric burns, migraines, and headaches.

## Complaints about Lake Alice in the 1970s

Oliver recalls that ACORD first got an inkling of what was occurring at Lake Alice on 1 December 1976 when Lyn Fry, a Department of Education psychologist, contacted him, worried about a 13-year-old boy Hakeagapuletama (Hake) Halo. Hake was from Niue and arrived in New Zealand at the age of 6 with his family. Like them, he spoke no English, and over the next few years, his language difficulties were often mis-identified as intellectual disability. Hake had engaged in some minor offending (shoplifting) and had a history of epilepsy, but no mental illness. Ms Fry had recommended that the Department of Social Welfare enrol him in Hokio Beach School (a residential school), but he was instead sent to Lake Alice, a decision that appalled her.

**Figure 1.**
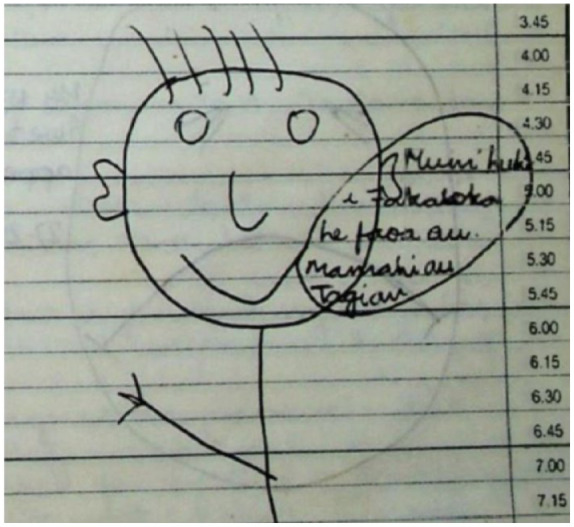
The drawing by Hake Halo depicted on the cover of the Royal Commission’s Beautiful Children report (Abuse in Care–Royal Commission of Inquiry, 2022). This is from one of six similar letters that Hake sent to his family. The translation from Niuean to English is ‘Mum I’m in pain and crying from electric shock’.

No one had explained to Hake’s family where he was being sent, or what might happen to him at the unit. Like many other children, soon after arrival, Hake was forcibly given painful paraldehyde injections and recriminatory electric shocks for perceived wrongdoings, without the knowledge and consent of his family. Hake needed an encrypted way to communicate his distress and pain to his mother, as staff were (unlawfully) screening the children’s’ correspondence to families. After earlier attempts to include a sad face were censored, Hake started drawing smiling faces, with speech bubbles containing his messages in Niuean, ‘I’ve been given electric shock by the people, mum. The pain is very bad’ said one*’* (Sutherland, 2020; Figure 1).

Within a fortnight of having heard about Hake’s situation from Ms Fry, ACORD had interviewed him and his family. They were horrified by what they learnt. This was the start of a long campaign for action. Initial communications to the Minster of Health were rebuffed, but contacting the NZ Herald, which published a damning story about Hake’s experiences at Lake Alice, garnered wider attention (Sutherland, 2020). The resulting Ministerial Inquiry essentially exonerated Leeks, an outcome abetted by the RANZCP, who spoke publicly in his favour. Around this time, the Citizen’s Commission on Human Rights (CCHR), founded by the Church of Scientology, also became involved, and they have been advocating for survivors since then.

In response to the publicity, more complainants emerged. ACORD documented their accounts and requested the Minister of Health close down the unit until they could be fully investigated. ‘If the allegations are proved, this misuse of the shock treatment will constitute perhaps the most appalling abuse of children in the guardianship of the state this country has known’ wrote Oliver in a 1977 press release (Sutherland, 2020).

Dr Leeks strenuously denied all allegations and claimed he had been a victim of a witch hunt. When asked during a media interview in 1977, whether he was shocking patients as a form of corporal punishment, he dismissed this suggestion as ‘arrant rubbish’ (p. 98, Abuse in Care–Royal Commission of Inquiry, 2022). He suggested the ‘bottom-of-the-barrel kids’ who said otherwise had been lying. A police investigation the next year failed to uphold the allegations and found no criminal misconduct, with supportive evidence from the RANZCP once more being influential (Sutherland, 2020).

## Dr Leeks leaves, and the unit closes

Despite not being formally censured, Dr Leeks’ reputation – and that of the unit – had been tarnished, and he resigned, leaving New Zealand to practice under the radar for a number of years in Australia. However, the spotlight was on him again in 2003, with *The Age* newspaper reporting Victorian Police were investigating complaints from several patients treated after he emigrated (The Age, 2003). In August 2006, Dr Leeks was ordered by an Australian court to pay a $55,000 in damages for sexually assaulting a former patient. The victim gave evidence that Dr Leeks told her complaining would be futile: ‘you’re a long-term psychiatric patient and no one will believe you’ (Johnston, 2006). Dr Leeks was now also under investigation from the Victorian Medical Board based on persisting allegations of his misconduct at Lake Alice. The past had caught up with him. He elected to surrender his medical registration rather than face the disciplinary hearing. However, he retained his membership of the RANZCP as a retired Fellow.

## The aftermath

The fight for justice for the children of Lake Alice has been a long one. Forty-six years have now passed since the first Magisterial Inquiry in 1977. In the intervening decades, survivors have sought redress through legal action, negotiation, public calls for inquiries, and complaints to NZ Police. The entities called upon included the Ombudsman, the Medical Association, the Medical Council, the RANZCP, the Department of Health, the Department of Education, the Department of Social Welfare, Cabinet, Crown Law, the Health and Disability Commissioner and the Accident Compensation Corporation. The Commission considered that none of the agencies contacted effectively investigated or held the perpetrators to account. From the outset there was sufficient evidence to justify criminal charges; as Solicitor-General Una Jagose later acknowledged, ‘the proof was right there in the file’ (p. 26, Abuse in Care–Royal Commission of Inquiry, 2022). The Commission’s report highlights failings in the police investigations in the 1970s and 2000s.

Conditional apologies and partial compensation were forthcoming in the 2000s. However, the Government did not acknowledge the long-term effects of the physical, sexual, and psychological abuse and torture. This lack of recognition led to applications to the UN Committee Against Torture by two survivors supported by the CCHR. The United Nations upheld these complaints, finding the children and been subjected to torture and urging NZ to conduct a full investigation and make redress (United Nations Committee Against Torture, 2020, 2022).

In 2021, the Police announced their intention to press charges based on evidence of criminal misconduct. However, due to their advanced age, poor health, and cognitive impairment, Selwyn Leeks and another former staff member, both in their 90s, were considered unfit to stand trial and so were not formally charged. Leeks died in Australia in January 2022. A further staff member was charged, but in June 2023, was found to be incapable of giving evidence in his own defence due to physical and mental impairment.

## Conclusions and implications for modern psychiatry

The Lake Alice Child and Adolescent Unit in New Zealand was intended to be a place of care and healing for vulnerable children and adolescents. It was anything but. Perhaps most horrifying is the sheer number of insiders who knew at the time of the abuse that took place within its walls and did nothing, and the outsiders who were told but responded only with incredulity and denial.

As a profession, psychiatry does not come across well based on its actions – and inactions – in the 1970s. The New Zealand branch of the RANZCP heard the allegations of Dr Leeks’ conduct while the unit was still operational, but did not forcefully advocate for change. Instead, perhaps doubtful that such things could be true, and spurred by defensiveness about electroconvulsive therapy and ‘anti-psychiatry’ sentiments, a number of consultants unquestioningly defended Dr Leeks. This was a significant missed opportunity that likely influenced the outcomes of the early inquiries and investigations in Dr Leeks’ favour.

Attempts to make some redress are underway currently, with the RANZCP consulting external experts, including the Commission’s Survivor Advisory Group of Experts and the Royal Commission Forum, for guidance on preparing and delivering a formal apology for these failings. In 2022, Tu Te Akaaka Roa (the NZ Committee) wrote to the RANZCP Board arguing that Dr Leeks’ misconduct was grounds for retrospective revocation of College membership according to the Constitution of the RANZCP. Although many years too late, Dr Leeks has now been posthumously stripped of his Fellowship; to the best of our knowledge, the first time this has occurred.

All NZ and Australian psychiatrists should read the executive summary if not the full report of *Beautiful Children*. Aaron Smale’s excellent podcast, *The Lake*, is also highly recommended (https://interactives.stuff.co.nz/2021/lake-alice-podcast/). The RANZCP might consider co-designing a teaching resource with people with lived experience on the abuses in psychiatry in Australasia. It is important that current and future psychiatrists understand it is not just temporally and politically distant places like Nazi Germany and Soviet Russia where State abuse can occur, and that everyone has a role in standing up and speaking out against the misuse of power. In June 2023, we held an educational session on Lake Alice for psychiatry registrars in the Lower North Island, which was very well received, and this model could be extended for a wider group of trainees.

The Commission’s full report and recommendations are due in March 2024. There will no doubt be further confronting truths. These too, as the Commission urges, should be faced head-on without excuses or explanations, but with a determination to ensure that such tragedies can never happen again.
